# Continuous Timescale Long-Short Term Memory Neural Network for Human Intent Understanding

**DOI:** 10.3389/fnbot.2017.00042

**Published:** 2017-08-23

**Authors:** Zhibin Yu, Dennis S. Moirangthem, Minho Lee

**Affiliations:** ^1^Department of Electrical Engineering, College of Information Science and Engineering, Ocean University of China Qingdao, China; ^2^School of Electronics Engineering, Kyungpook National University Daegu, South Korea

**Keywords:** continuous timescale, recurrent neural network, LSTM, classification, dynamic sequence

## Abstract

Understanding of human intention by observing a series of human actions has been a challenging task. In order to do so, we need to analyze longer sequences of human actions related with intentions and extract the context from the dynamic features. The multiple timescales recurrent neural network (MTRNN) model, which is believed to be a kind of solution, is a useful tool for recording and regenerating a continuous signal for dynamic tasks. However, the conventional MTRNN suffers from the vanishing gradient problem which renders it impossible to be used for longer sequence understanding. To address this problem, we propose a new model named Continuous Timescale Long-Short Term Memory (CTLSTM) in which we inherit the multiple timescales concept into the Long-Short Term Memory (LSTM) recurrent neural network (RNN) that addresses the vanishing gradient problem. We design an additional recurrent connection in the LSTM cell outputs to produce a time-delay in order to capture the slow context. Our experiments show that the proposed model exhibits better context modeling ability and captures the dynamic features on multiple large dataset classification tasks. The results illustrate that the multiple timescales concept enhances the ability of our model to handle longer sequences related with human intentions and hence proving to be more suitable for complex tasks, such as intention recognition.

## Introduction

In machine learning, dynamic sequence modeling is a burning research topic, which includes intention understanding, action recognition, language understanding, semantic understanding (Peniak et al., 2011; Wasser and Lincoln, 2012; Wonmin et al., 2015; Kim et al., 2017) etc. Unlike popular static models, such as Convolutional Neural Network (CNN) (LeCun et al., 1998) and Deep Belief Network (DBN) (Hinton and Salakhutdinov, 2006) that focus on the feature of the data without considering any time dependency, the dynamic models try to find the relationships between data following the time axis. Context, which is generally mentioned in language understanding (Ghadessy, 1999; Givón, 2005), also plays an important role in dynamic sequence classification. Context contains several physical and abstract aspects such as time, symbols, location, names, etc. to describe the background of dynamic signal. Same words may have different meaning under different contexts. In short, context plays the role of surroundings, which contains some inconspicuous but important descriptions of the current phenomenon. Context can be deemed as the key of the dynamic sequence learning.

Multiple Timescales Neural Network (MTRNN), developed by Tani et al. (2008), is believed to be efficient to hold the context of dynamic trajectories. MTRNN is a successive extension of Recurrent Neural Network (RRN). All biological neural networks are recurrent (Jaeger, 2002), which is one of the reasons to choose RNN for dynamic sequence modeling. MTRNN, in turn, consists of multiple Continuous Recurrent Neural Network (CTRNN) layers. Each CTRNN layer is allowed to have one or more different timescale constants. Different time constants imply different activation speeds. That is why this network is called “multiple timescales.” Inspired by the structure of human brain, MTRNN has been proved to be useful on goal-planning problems (Arie et al., 2009; Jeong et al., 2012).

There are several extensions of RNN such as Elman networks, Jordan network, etc. These extensions aim to improve the memory ability and the performance of RNN (Cruse, 2006) but suffer from the vanishing gradient problem (Hochreiter et al., 2001). Long Short Term Memory network (LSTM), developed by Hochreiter and Schmidhuber (1997), and promises to overcome this problem. Similar to most RNNs, LSTM also uses derivative based methods to evolve itself. LSTM uses several gates with different functions to control the neurons and store the information. LSTM cell has the ability to keep important information for a longer period it is used. This property of holding information allows LSTM to perform well on classifying, processing or predicting a complex dynamic sequence. Research has shown that LSTM can achieve better performance than Hidden Markov Model (HMM) along with other RNNs on several real-world problems, such as handwriting recognition (Graves and Schmidhuber, 2005; Baccouche et al., 2011; Graves et al., 2013). It has also been proved that RNN performs well in human action modeling (Schrodt and Butz, 2016; Bütepage et al., 2017a). Moreover, deep RNN structures are able to represent human motion and natural language (Bütepage et al., 2017b; Plappert et al., 2017). Thus, deep RNN is a good candidate to handle human motion and language modeling problems. But how to design an efficient deep RNN structure is still a challenging problem.

We intend to capture the context efficiently while overcoming the vanishing gradient problem, which is still existing in CTRNN and MTRNN. We propose a model considering the advantages of an LSTM and inheriting the biological idea given by CTRNN. The proposed Continuous Timescale Long-Short Term Memory (CTLSTM) builds a temporal hierarchy into the architecture that enhances the model's ability to solve long-term complex sequence modeling problems. We evaluate our model on multiple public datasets to compare with the baselines. We demonstrate the capability of our model in human action classification tasks as well as human intention recognition tasks which consist of longer multiple action sequences. Our results illustrate that our proposed model outperforms the existing baselines.

The remainder of this paper is organized as follows: The proposed model is described in Section Proposed Model. The experiments and results are reported in Section Experiments and Results. Finally, the conclusion and discussion are presented in Section Conclusion and Discussion.

## Proposed model

We describe the proposed model in this section including the background study as well as the motivation of our model.

### Motivation

Dynamic sequence, in general, is a number set (vector) combination in which each vector has a given time or spatial coordinates. A dynamic model can also be considered as a set of relationships between two or more measurable quantities. It relies on one or more fixed rules to describe how the dynamic model works and evolves itself. At any given point of time, a dynamic system has a state given by a set of real numbers (a vector) that can represent the current situation.

Inspired by MTRNN and LSTM, we aim to develop a RNN with multiple timescales structure with better ability to capture the dynamic features in longer sequences such as a series of human actions for understanding human intention. Time constants, which are the key of CTRNN, can be defined separately for each neuron node. Different time constants lead to different neuron activation abilities. For example, neurons with large timescale will activate slowly. That means slow neuron will become inactive to some short-term signals. Once the neuron starts firing, it would last for a longer time according to its timescale. Based on the results of previous research (Tani et al., 2008; Arie et al., 2012; Jeong et al., 2012; Yu and Lee, 2015a,b), we believe that different time scales would bring benefits for dynamic signal modeling. Thus, to inherit the advantages of MTRNN, the model is designed with different time scales. Layers with different time constants work differently. Layers with slower time constants would focus on signal organization and planning, while layers with faster time constants can implement the elemental dynamic sequences. Yu and Lee (2015a) and Kim et al. (2017) have already demonstrated the use of MTRNN in motion based intent recognition tasks. On the other hand, LSTM, which has a more complex structure than the common RNN neuron, is efficient in various applications involving long-term dependency (Gers et al., 2000, 2002). We aim to design a dynamic system, which has the multiple timescales structure but with more efficient neurons.

### Continuous timescale recurrent neural network

**Figure 1:**
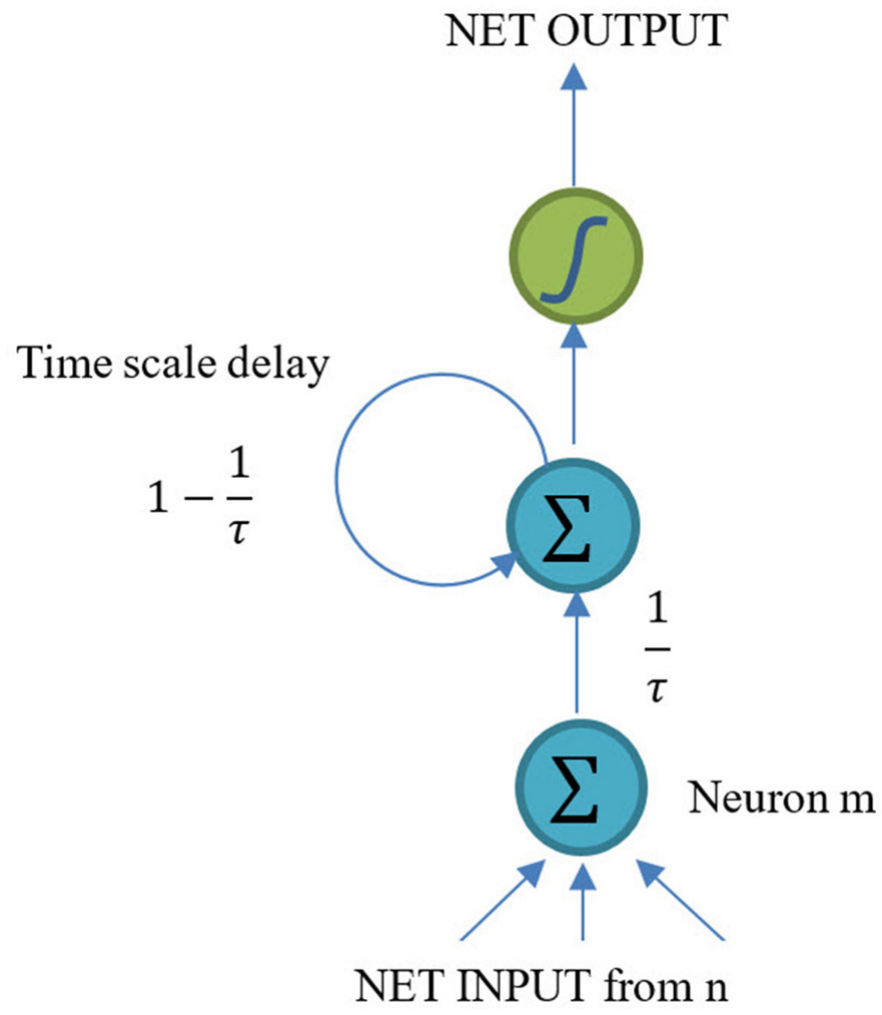
Structure of a CTRNN neuron.

CTRNN, which is also an extension of RNN, is a kind of artificial neural network described by Hopfield, Tank, and Beer (Hopfield and Tank, 1986; Beer, 1995). With a plausible biological interpretation and inexpensive computational complexity, CTRNN has always been used to explain biological phenomena (Kier et al., 2006). The structure of CTRNN is shown in Figure 1.

CTRNNs were developed by Beer (1995). The basic hypothesis is:

(1) $$\tau_{m}\frac{dy_{m}}{dt}=-y_{m}^{t}+\sum_{n}^{N}w_{nm}\theta \left(y_{n}^{t}-b_{n}\right)+I_{i}(t)$$

where τ_*m*_ is the membrane time constants of the neuron *m*; $y_{m}^{t}$ is the membrane potential after the deletion of the action potential; *b*_*n*_ is the bias of the neuron *n* (*n* ∈ *N*); *I*_*i*_(*t*) is the additional input in time *t*; θ(.) is the activation function which could be logistic sigmoid, softmax or hyperbolic tangent.

Equation (1) was derived based on the RC circuit neural model (Dwyer et al., 2010). Thus, CTRNN has a clear interpretation rule from the biological neurons to the artificial neurons of the engineering model. For this very reason, CTRNNs have been used to explain biological phenomenon.

Similar to RNN, the forward process of CTRNN can be concluded as:

(2) $$u_{m}^{t}=\left(1-\frac{1}{\tau_{m}}\right)u_{m}^{t-1}+\frac{1}{\tau_{m}}\left(\sum_{i}^{I}w_{im}x_{i}^{t}+\sum_{h}^{H}w_{hm}y_{h}^{t-1}\right)$$

where τ_*m*_ is the time constant of the neuron *m*; $u_{m}^{t}$ is the presynaptic value of the *m*^*th*^ neuron in the *t*^*th*^ step and *x* is the net inputs of the neuron *m*; *w*_*hm*_ is the weight between the *h*^*th*^ neuron to the *m*^*th*^ neuron; *I* represents the direct inputs of neuron *m* and *H* denotes all other hidden neurons with have weight connections to *m*. After the presynaptic values are obtained, the activation output can be calculated with suitable activation function. The importance of τ_*m*_ is to produce a resistance to reject the input from other neurons and try to keep the history information in the neuron. Larger τ_*m*_ means stronger resistance and a slower activation process. In other words, a neuron with large time constant attempts to store the history information and needs a longer time to accept new inputs.

Back Propagation Through Time (BPTT) can also be used to update the weights of CTRNN as:

(3) $$\begin{array}{l} \frac{\partial E}{\partial u_{m}^{t}}=\theta^{\prime }\left(u_{m}^{t}\right)\left(\sum_{o}^{O}w_{mo}\frac{\partial E}{\partial u_{o}^{t}}+\frac{1}{\tau_{h}}\sum_{h}^{H}w_{mh}\frac{\partial E}{\partial u_{h}^{t+1}}\right)+\\ \;\left(1-\frac{1}{\tau_{m}}\right)\frac{\partial E}{\partial u_{m}^{t+1}} \end{array}$$

where τ_*h*_ is the time constant of the neuron *h*; *O* denotes the output neurons; $\frac{\partial E}{\partial u_{m}^{t}}$ represents the error gradient of the neuron $u_{m}^{t}$. Please note that τ_*m*_ and τ_*h*_ can be different. With the derivative and the synaptic outputs, weights between two neurons can be obtained using Equation (4).

(4) $$\frac{\partial E}{\partial w_{mn}}=\sum_{t}^{T}\frac{\partial E}{\partial u_{n}^{t}}\frac{\partial u_{n}^{t}}{\partial w_{mn}}=\sum_{t}^{T}\frac{\partial E}{\partial u_{n}^{t}}y_{m}^{t}$$

### Long-short term memory

LSTM was created by Hochreiter and Schmidhuber (1997). Unlike the previous RNN models (mentioned in Section Continuous Timescale Recurrent Neural Network) that focus on biological interpretation, LSTM was developed as an engineering model to solve the vanishing gradient problem (Hochreiter et al., 2001).

**Figure 2:**
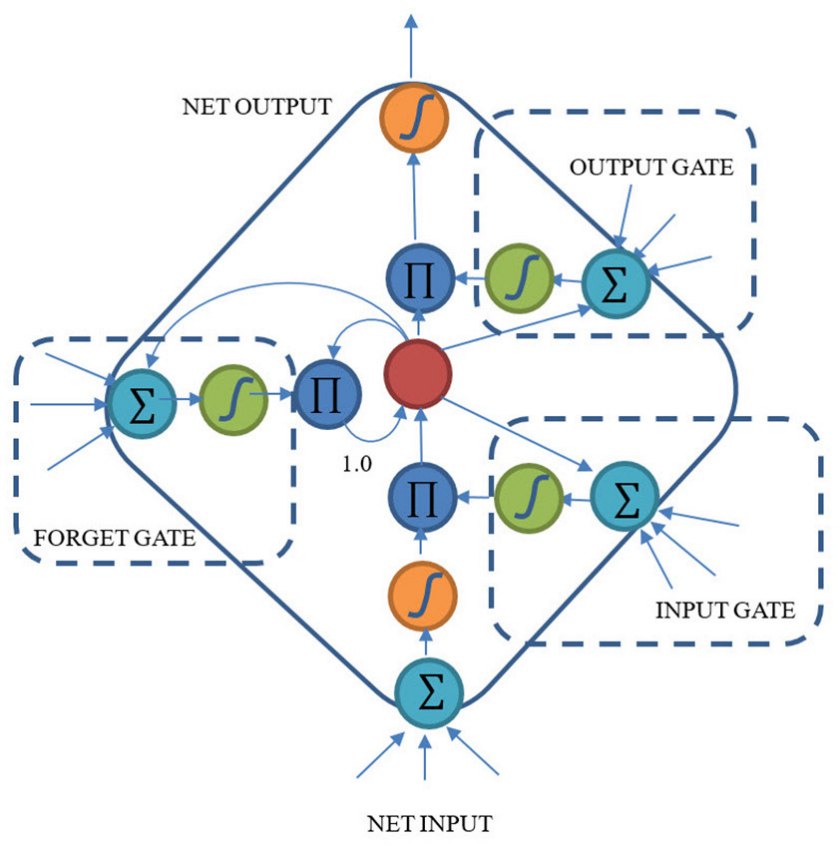
Structure of LSTM block with a single cell.

The structure of LSTM is shown in Figure 2. In order to solve the vanishing gradient problem, the first model of LSTM defines two kinds of gates: input and output gates. Input gate is used to control whether the cell should accept the input information or not. The output gate decides whether the cell should output the contents stored in the cell. Gers et al. improved this prototype and added a forget gate to the model in 2000 (Gers et al., 2000). The forget gate provides a way to reset the contents of cells. LSTM was further improved by Gers et al. (2002). They added the peephole connections to make it possible for the cells to control the time for gate opening inside the block.

The LSTM cells are key in handling the vanishing gradient problem. LSTM can control the information though time and can retain the important information by making the information flow unchanged all along the time steps. LSTM has the ability to add or remove information via the three gates and each gate learns to do so through backpropagation.

### The proposed continuous timescale long-short term memory (CTLSTM) model

As shown in Figure 1, CTRNN neuron is still very similar to a traditional RNN neuron. The difference between a CTRNN neuron and an RNN neuron is that the CTRNN neuron considers a time scale delay after calculating the network input. However, LSTM uses a considerably different structure called block and cells instead of the traditional RNN neurons. An LSTM block includes three different gates and several cells (Only one cell is shown in Figure 2). Each cell has an input and an output. But the same gates control cells in one block. The inputs of gates are similar with net inputs. Both direct inputs and neuron (block) outputs from other hidden layer could be the gate inputs or net inputs. Although these two structures are quite different from each other, the input and output rules are still similar. This makes it possible for us to combine these two models.

**Figure 3:**
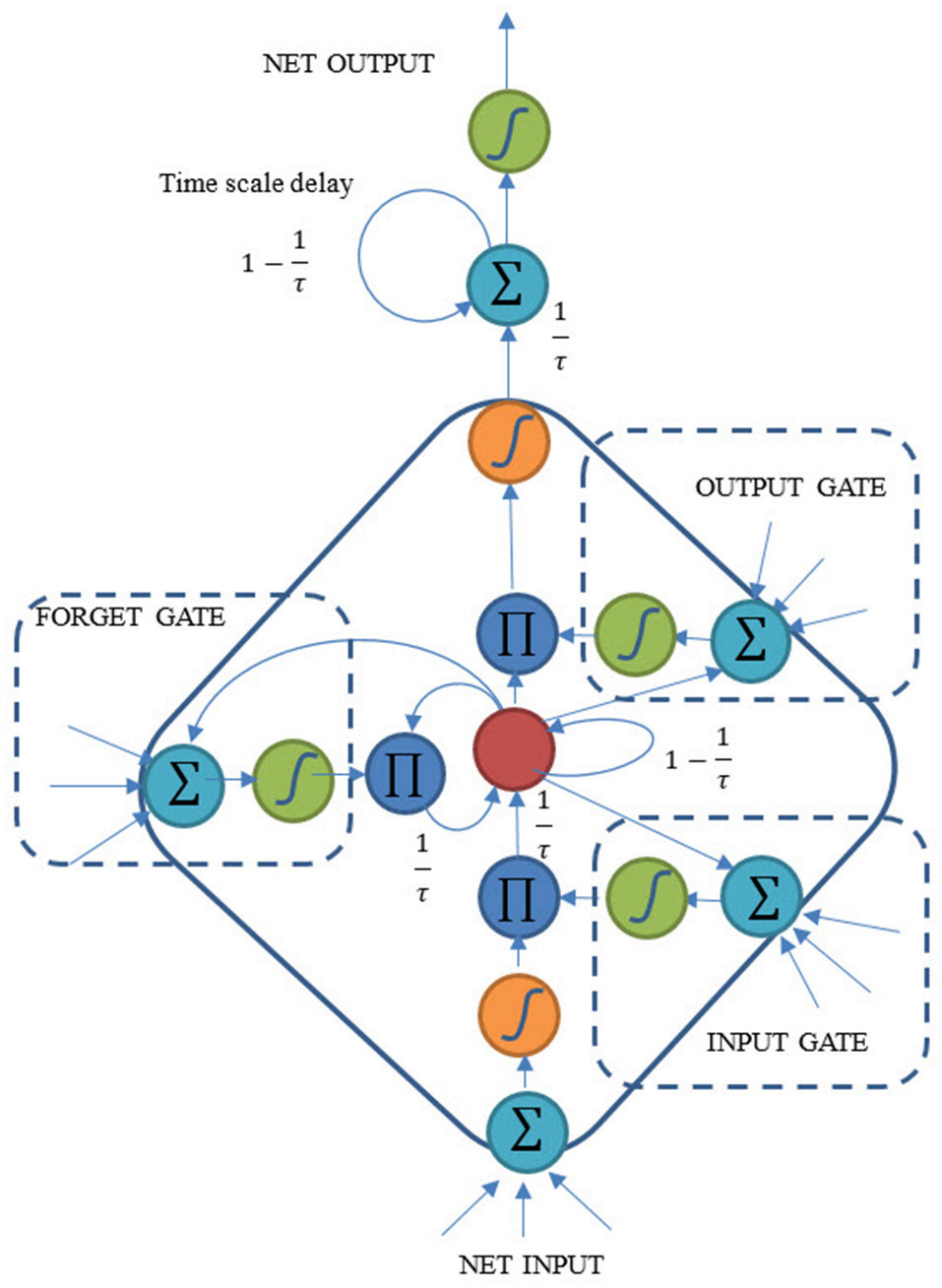
Structure of the proposed Continuous Timescale LSTM (CTLSTM).

The proposed CTLSTM model is shown in Figure 3. It is known that LSTM cell is able to capture the non-linear properties and can solve the “vanishing gradient” problem. The idea of CTLSTM network is to separate different tasks to different blocks with different timescales. We integrate the LSTM model with the CTRNN model by including a timescale delay at the end of the block. This idea has been proved to be efficient in the case of MTRNN (Alnajjar et al., 2013). The forward process of the proposed CTLSTM model is shown in Equations (5–13).

(5) $$u_{l}^{t}=\sum_{i}^{I}w_{im}x_{i}^{t}+\sum_{h}^{H}w_{hm}y_{h}^{t-1}+\sum_{c}^{C}w_{cm}s_{c}^{t-1}$$

(6) $$y_{l}^{t}=f(u_{l}^{t})$$

(7) $$u_{\phi }^{t}=\sum_{i}^{I}w_{i\phi }x_{i}^{t}+\sum_{h}^{H}w_{h\phi }y_{h}^{t-1}+\sum_{c}^{C}w_{c\phi }s_{c}^{t-1}$$

(8) $$y_{\phi }^{t}=f(u_{\phi }^{t})$$

(9) $$u_{c}^{t}=\sum_{i}^{I}w_{ic}x_{i}^{t}+\sum_{h}^{H}w_{hw}y_{h}^{t-1}$$

(10) $$s_{c}^{t}=y_{\phi }^{t}s_{c}^{t-1}+y_{l}^{t}g(u_{l}^{t})$$

(11) $$u_{w}^{t}=\sum_{i}^{I}w_{iw}x_{i}^{t}+\sum_{h}^{H}w_{hw}y_{h}^{t-1}+\sum_{c}^{C}w_{cw}s_{c}^{t}$$

(12) $$y_{w}^{t}=f(u_{w}^{t})$$

(13) $$y_{c}^{t}=\frac{1}{\tau }y_{w}^{t}h\left(s_{c}^{t}\right)+(1-\frac{1}{\tau })y_{c}^{t-1}$$

The activation process of the input gate is shown in Equations (5) and (6). *i, h*, and *c* denote the input, hidden and cell state, respectively. Similarly, forget gate is represented in Equations (7) and (8). Cell input is obtained in Equation (9) and the cell state is calculated using Equation (10). Similar to input and forget gate, the output gate activation function is represented in Equations (11) and (12). States at time *t* step $s_{c}^{t}$ are used for the input of the output gate in time *t*, while the state in *t* - 1 step $s_{c}^{t-1}$ is used for calculating the input and forget gate values in time *t*. Finally, the cell outputs are calculated using Equation (13) where we added a time constant τ for each cell. Larger τ means slower cell outputs, and can make the cell focus on the slow features of the dynamic input signal. The traditional LSTM block would be a special case of CTLSTM when τ = 1. *f*(.) is the activation function of the gates while *g*(.) and *h*(.) are the activation function of the cell input and output, respectively. We followed Graves and Schmidhuber (2005) and define *f*(.) as logistic sigmoid function while *g*(.) and *h*(.) are hyperbolic tangent functions.

According to the forward process (Equations 5–13) and the BPTT rules, the backward pass (Equations 14–18) can be derived as:

(14) $$\begin{array}{l} \frac{\partial E}{\partial y_{c}^{t}}=\left(1-\frac{1}{\tau }\right)\frac{\partial E}{\partial y_{c}^{t+1}}+\sum_{k}^{K}w_{ck}\frac{\partial E}{\partial u_{k}^{t}}+\\ \sum_{h}^{H}w_{ch}\frac{\partial E}{\partial u_{h}^{t+1}} \end{array}$$

(15) $$\frac{\partial E}{\partial u_{w}^{t}}=\frac{1}{\tau }f^{\prime }(u_{w}^{t})\sum_{c}^{C}h(s_{c}^{t})\frac{\partial E}{\partial y_{c}^{t}}$$

(16) $$\begin{array}{l} \frac{\partial E}{\partial s_{c}^{t}}=\frac{1}{\tau }y_{w}^{t}h^{\prime }(s_{c}^{t})\frac{\partial E}{\partial u_{co}^{t}}+y_{\phi }^{t+1}\frac{\partial E}{\partial s_{c}^{t+1}}+w_{cl}\frac{\partial E}{\partial u_{l}^{t+1}}\\ +w_{c\phi }\frac{\partial E}{\partial u_{\phi }^{t+1}}+w_{cw}\frac{\partial E}{\partial u_{w}^{t}} \end{array}$$

(17) $$\frac{\partial E}{\partial u_{c}^{t}}=f(u_{\phi }^{t})\;\frac{\partial E}{\partial s_{c}^{t}}$$

(18) $$\frac{\partial E}{\partial u_{\phi }^{t}}=f^{\prime }(u_{\phi }^{t})\sum_{c}^{C}s_{c}^{t-1}\frac{\partial E}{\partial y_{c}^{t}}$$

(19) $$\frac{\partial E}{\partial u_{l}^{t}}=f^{\prime }(u_{l}^{t})\sum_{c}^{C}g(u_{c}^{t})\frac{\partial E}{\partial y_{c}^{t}}$$

where the derivative of cell outputs are calculated in Equation (14), $\;\sum_{k}^{K}w_{ck}\frac{\partial E}{\partial u_{k}^{t}}$ is the error term from the output layer and $\sum_{h}^{H}w_{ch}\frac{\partial E}{\partial u_{h}^{t\pm 1}}$ denotes the error come from other hidden layers. *H* can be cell, gate or the neurons of the RNN. Equations (15), (18), and (19) represent the error term of output gate, forget gate, and input gate, respectively. The cell state error is calculated in Equation (16) and cell input error is shown in Equation (17).

**Figure 4:**
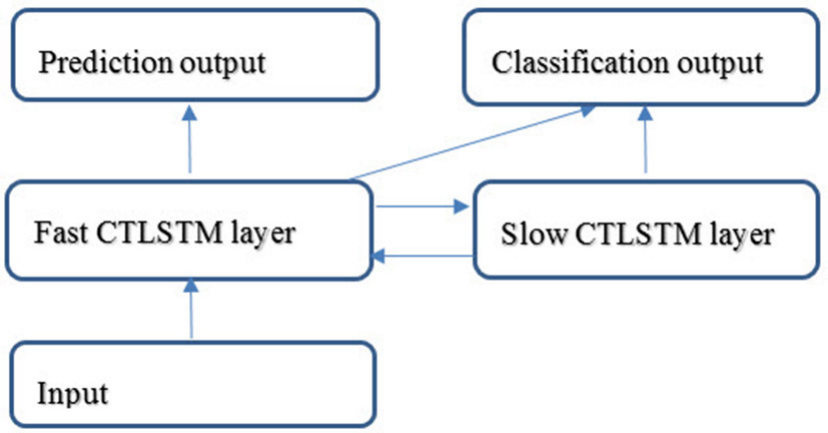
Structure of a CTLSTM network.

Figure 4 shows an application example of the proposed CTLSTM network. We use two CTLSTM layers to build a CTLSTM model. Similar to Supervised MTRNN (Yu and Lee, 2015a), CTLSTM also has slow and fast context layers and can work for both classification and prediction tasks simultaneously. We believe that the fast CTLSTM layer can focus on the fast fractional work while slow CTLSTM can work for slow organizing tasks. This property will help the CTLSTM model to capture the dynamic context from the longer sequences efficiently.

## Experiments and results

In order to evaluate our model, we conducted several experiments using multiple datasets including human motion and intention recognition. The mean results are reported with ± s.d. for the performance over 10 runs for each task. We also report the Wilcoxon signed-rank statistical test results to find the significance of the performance of CTLSTM over the existing model in each task. The details of each experiment and the results are illustrated in this section.

### UCI character trajectories dataset

**Figure 5:**
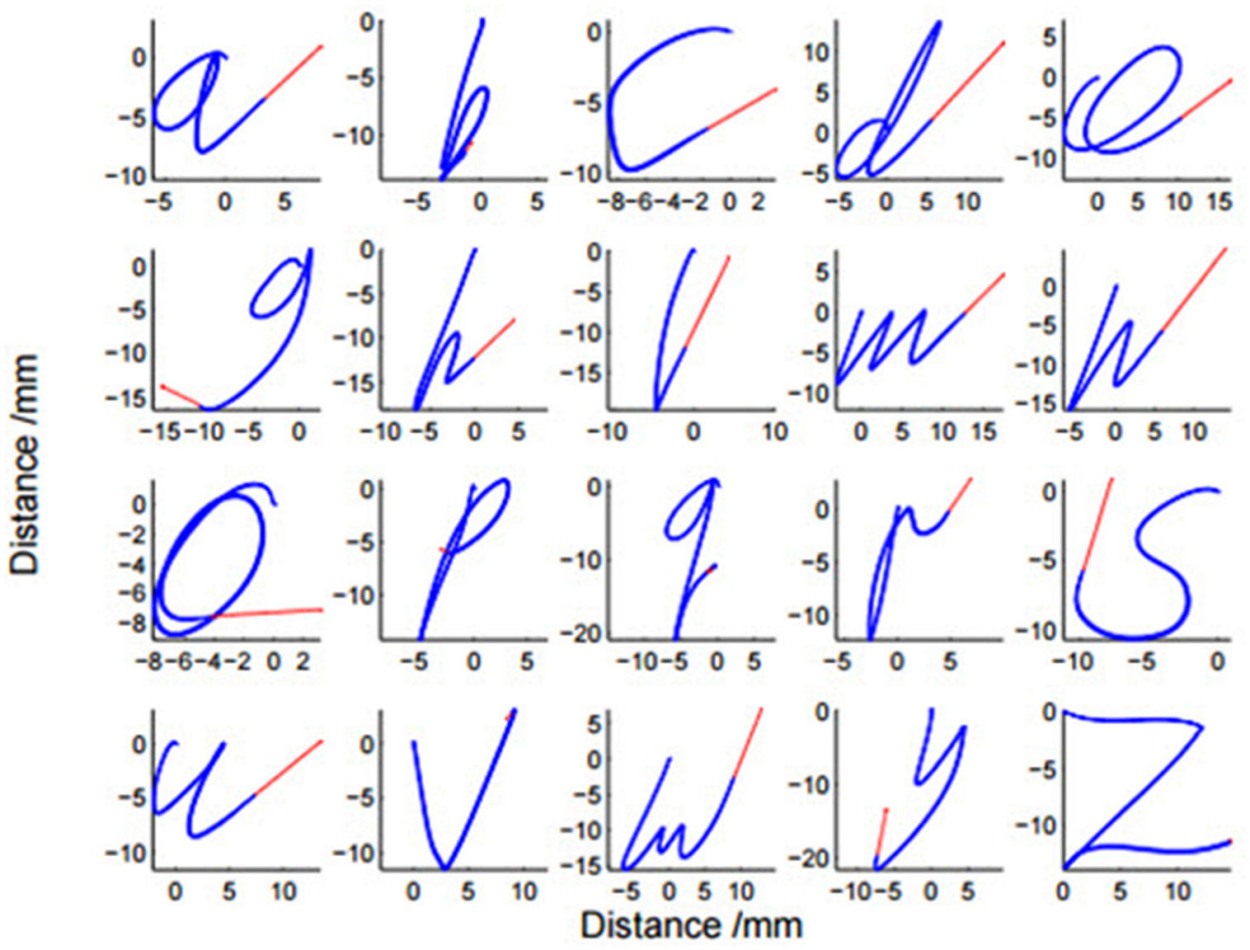
Twenty samples of characters taken from the mixed dataset. Total size of dataset is 2,858 characters, with over 100 samples of each character.

We used the character trajectories dataset which is a part of the UCI dataset (Williams et al., 2006). It has a total of 2,858 samples and 20 kinds of character trajectories. The data consist of three dimensions which is *x, y*, and the pen tip force. This dataset consists of only one stroke characters with a single “PEN-DOWN” segment since the character segmentation was performed using a pen tip force cut-off point. For example, characters like “*t*” or “*f* ” were not included in the dataset. The details of the 20 kinds of characters are shown in Table 1 and Figure 5. 1,433 randomly selected samples are used for training and the remaining 1,425 samples are used for testing. We train the CTLSTM and LSTM models for 500 epochs. This stopping point was chosen since the error does not decrease after an additional training of 50 epochs. The neuron cell states are initialized as set as 0 in all experiments.

**Table 1:** UCI dataset description.

**Train and test sequences**			
**Classes**	**Training set**	**Test set**	**Total**
a	97	74	171
b	73	68	141
c	55	87	142
d	82	75	157
e	113	73	186
g	66	72	138
h	57	70	127
l	80	94	174
m	69	56	125
n	56	74	130
o	68	73	141
p	70	61	131
q	70	54	124
r	57	62	119
s	64	69	133
u	67	64	131
v	74	81	155
w	60	65	125
y	67	70	137
z	88	83	171
Total	1,433	1,425	2,858

The learning rate for both the baseline model using LSTM and the proposed model using CTLSTM was set to 0.00001. 130 blocks (100 + 30 as two layers) were used in the LSTM model. 100 fast CTLSTM blocks (τ = 1) and 30 slow CTLSTM blocks were used to build the CTLSTM model. Each block of CTLSTM or LSTM contain only one cell. For a fair comparison, we chose the same network structure (100 + 30 as two layers) as described in Figure 4 for both CTLSTM and LSTM. Max pooling was used for classification decision in both models. Both offline and online classification results are shown in Table 2. The definition of offline and online accuracy are given below:

(20) $$P(x)=\frac{N_{x}}{N_{total}}$$

(21) $$Acc_{offline}^{c}=\{\begin{array}{ll} 1\;if\;\underset{x}{\text{argmax}}P(x) & =\;C\\ 0 & else \end{array}$$

(22) $$Acc_{online}^{c}=P(c)=\frac{N_{c}}{N_{total}}$$

(23) $$Acc_{weighted\;avg}=\sum_{m}^{M}\frac{N_{m}}{\sum_{m}^{M}N_{m}}Acc_{online}^{m}$$

where *N*_*x*_ is the frame number which is classified as class *x* and *N*_*total*_ is the total frame number of the current sequence. In simple terms, there are only two cases of offline classification on one sample: 100 or 0%. But online classification requires a real-time per frame accuracy. Since, this dataset is unbalanced, we performed a weighted average according to Equation (27), where *N*_*m*_ is the frame number of class *m*, *M* is the species number and $Acc_{online}^{m}$ is the online accuracy of class *m*.

**Table 2:** Classification results for UCI dataset.

**Accuracy (True positive)**				
	**Max-pooling (Offline)**		**Real-time (Online)**	
**Classes**	**CTLSTM**	**LSTM**	**CTLSTM**	**LSTM**
a	75.68 ± 15.02	45.14 ± 19.35	45.59 ± 5.49	35.14 ± 9.73
b	87.65 ± 13.59	80 ± 9.54	72.94 ± 9.73	64.22 ± 8.28
c	87.36 ± 7.69	68.85 ± 10.87	53.88 ± 5.01	42.58 ± 6.63
d	98.8 ± 2.1	97.6 ± 3.31	78.68 ± 4.09	74.7 ± 2.68
e	97.26 ± 0.61	96.44 ± 4.21	90.22 ± 1.49	89.51±.3.97
g	97.92 ± 1.55	94.17±.4.68	78.4 ± 3.28	72.07 ± 6.8
h	83 ± 8.89	75.86 ± 9.46	66.87 ± 7.75	62.02 ± 8.15
l	92.34 ± 14.55	60.96 ± 16.8	71.49 ± 5.92	54.1 ± 14.02
m	74.82 ± 8.54	79.29 ± 11.01	67.81 ± 7.1	68.63±.9.19
n	53.78 ± 10.02	42.43 ± 12.27	43.57 ± 7.62	32.48 ± 8.54
o	76.99 ± 10.1	73.97 ± 11.15	54.79 ± 7.36	57.16 ± 8.32
p	94.59 ± 3.03	93.44 ± 5.96	77.69 ± 2.37	73.7 ± 6.48
q	92.22 ± 3.78	81.11 ± 10.37	70.02 ± 5.3	65.43 ± 9.08
r	60 ± 12.38	70.48 ± 18.18	55.64 ± 7.51	62.28 ± 15.27
s	96.52 ± 5.55	96.67 ± 2.25	79.49 ± 5.55	78.62 ± 2.99
u	79.06 ± 10.69	80.47 ± 8.16	60.79 ± 7.99	59.26 ± 6.47
v	98.64 ± 2.94	98.77 ± 2.34	81.24 ± 2.76	81.96 ± 2.63
w	73.54 ± 9.23	85.85 ± 6.8	62.21 ± 7.23	70.77 ± 5.76
y	93.86 ± 3.62	96.14 ± 3	71.95 ± 4.59	75.06 ± 3.57
z	100 ± 0	100 ± 0	95.33 ± 2.42	95.17 ± 1.6
**Avg**.	**85.7** ± **1.53**	80.88 ± 3.74	**68.93** ± **0.98**	65.74 ± 2.54
**Weighted Avg**.	**86.2** ± **1.72**	80.57 ± 3.75	**69.12** ± **1.02**	65.5 ± 2.49

*Numbers in bold show the best performance in the particular task*.

Theoretically, the timescale should be similar to the length of a dynamic feature. From **Figure 7** we can find that a dynamic feature (e.g., from peak to valley) ranges from 10 to 30 frames, thus we chose 20 as the slow context timescale for this task.

**Figure 6:**
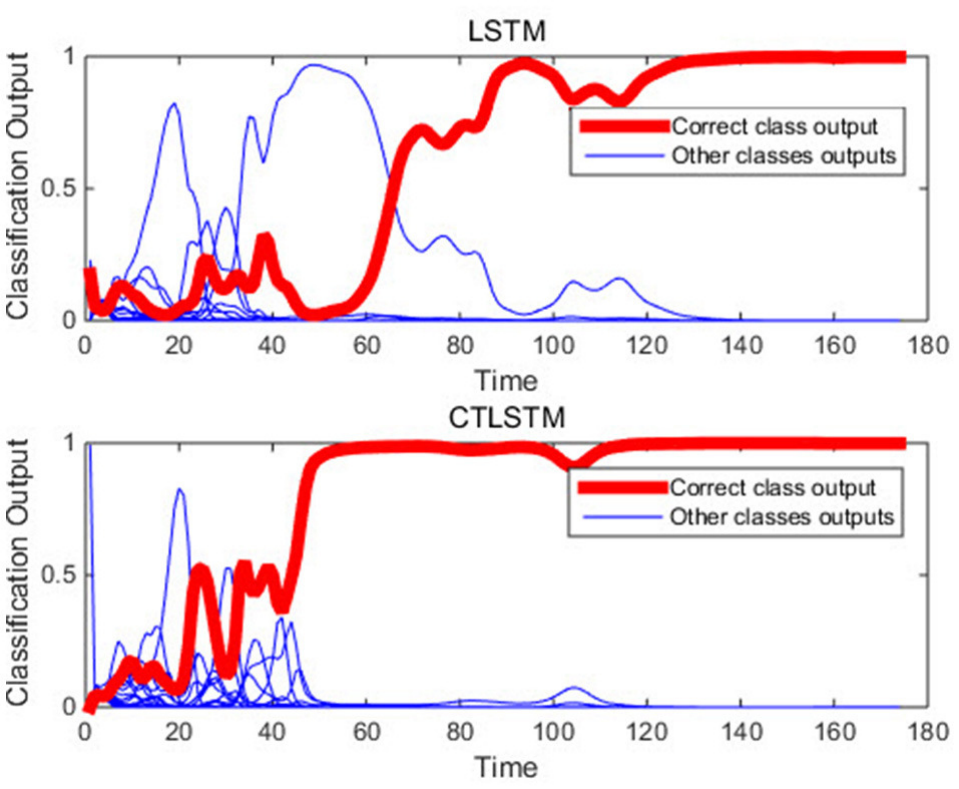
An example of real-time classification outputs of the two models (character m).

**Figure 7:**
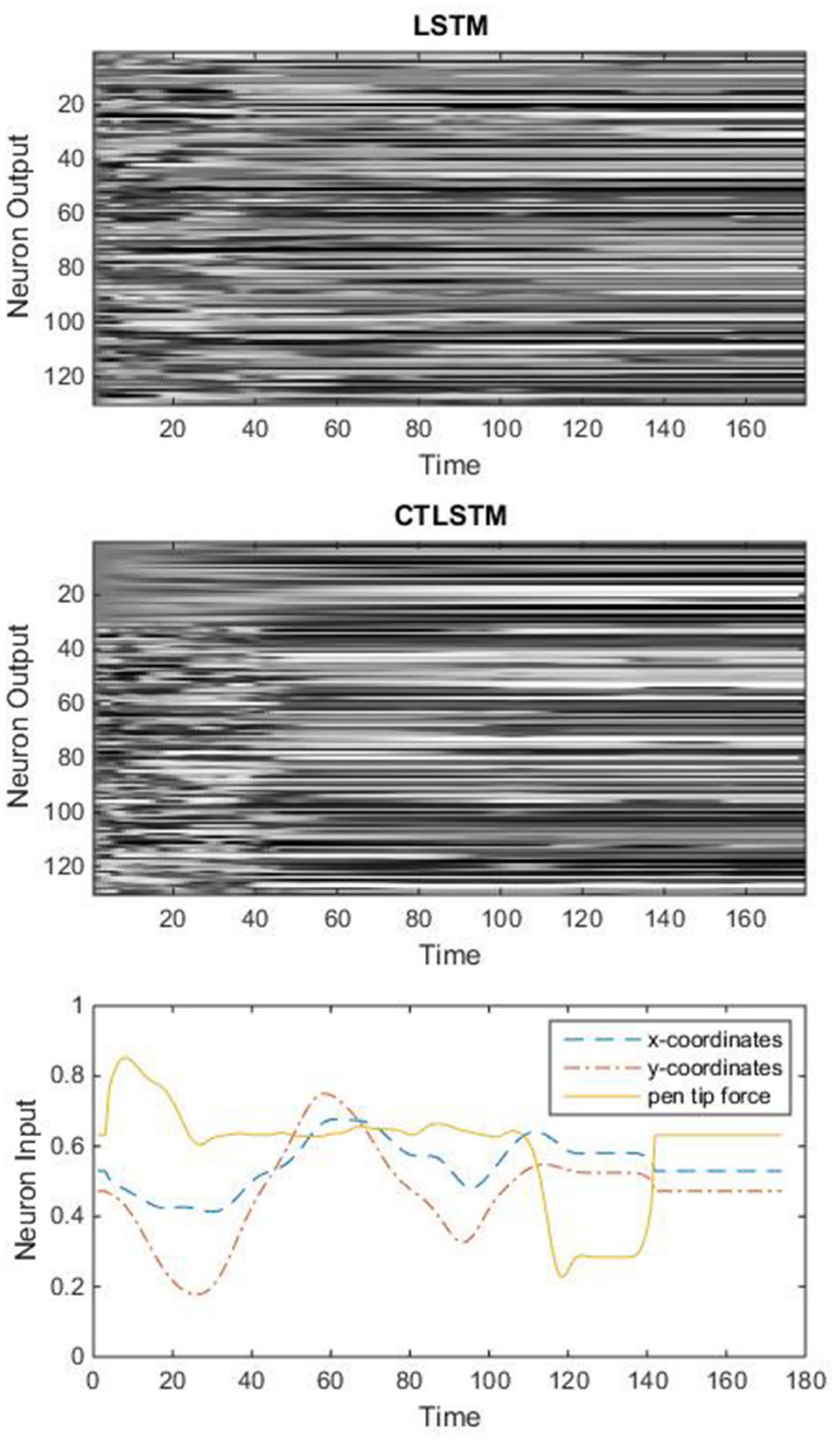
An example of real-time neuron activation of the two models (character m).

**Figure 8:**
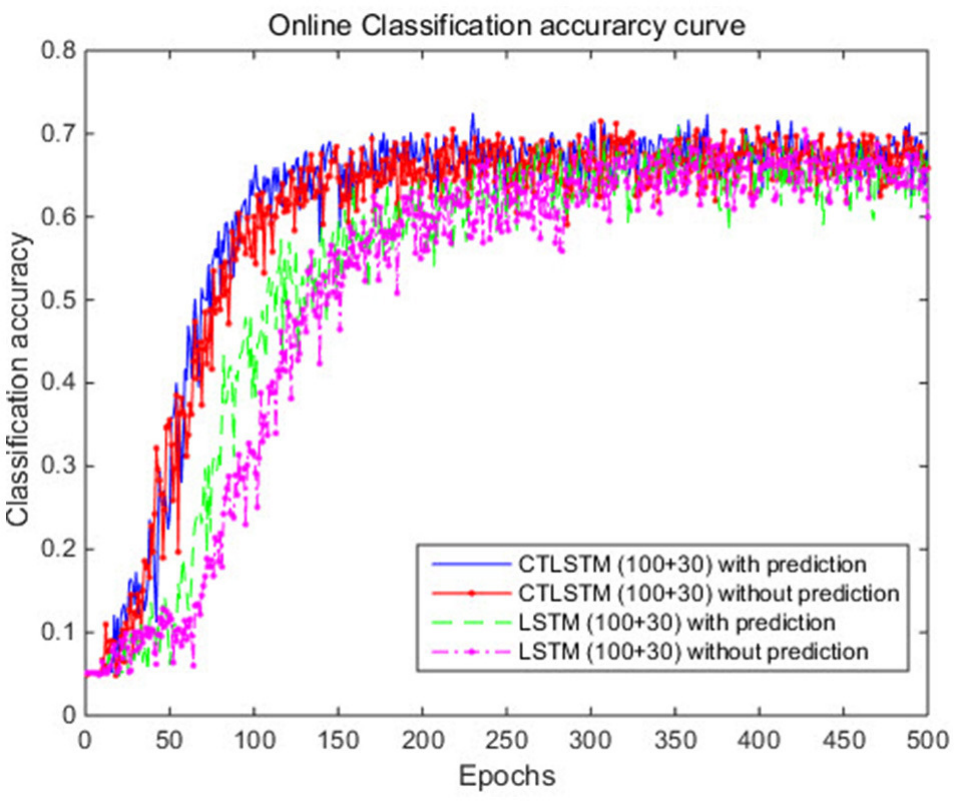
Classification accuracy (true positive) curve of CTLSTM and LSTM for UCI dataset.

**Figure 9:**
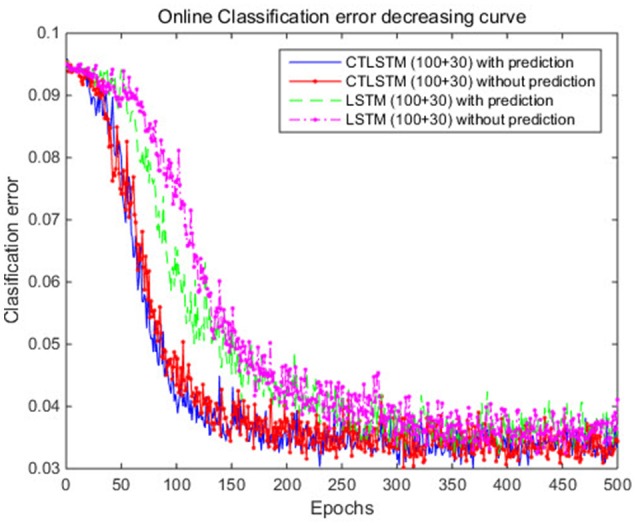
Classification error decreasing curve of CTLSTM and LSTM for UCI dataset.

**Figure 10:**
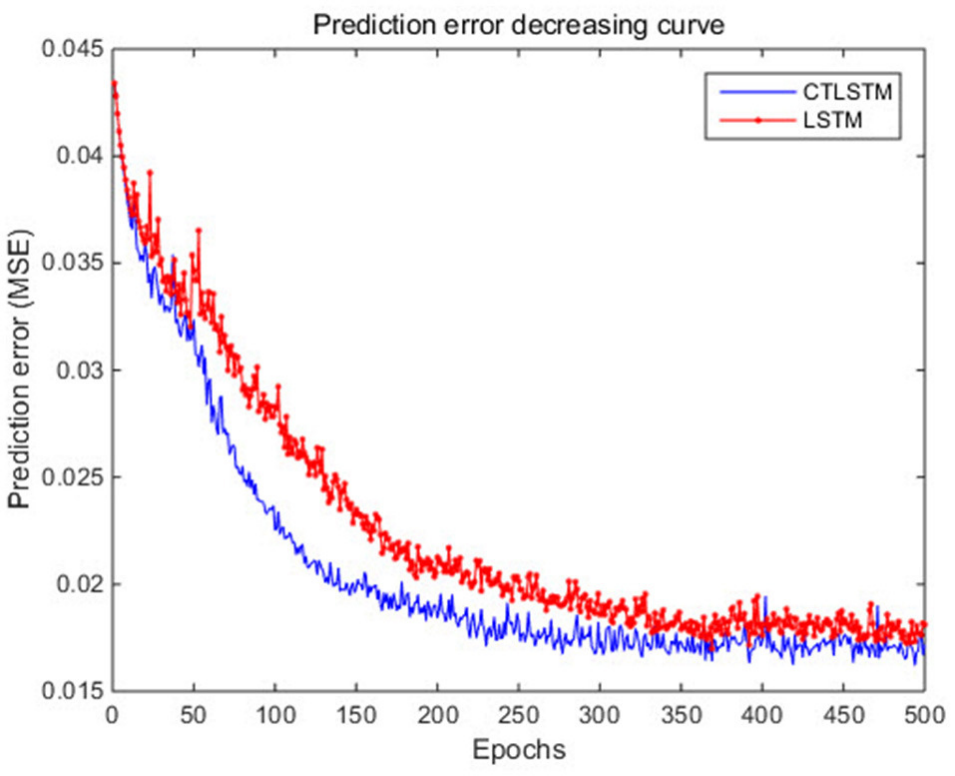
Prediction error decreasing curve of CTLSTM and LSTM for UCI dataset.

**Figure 11:**
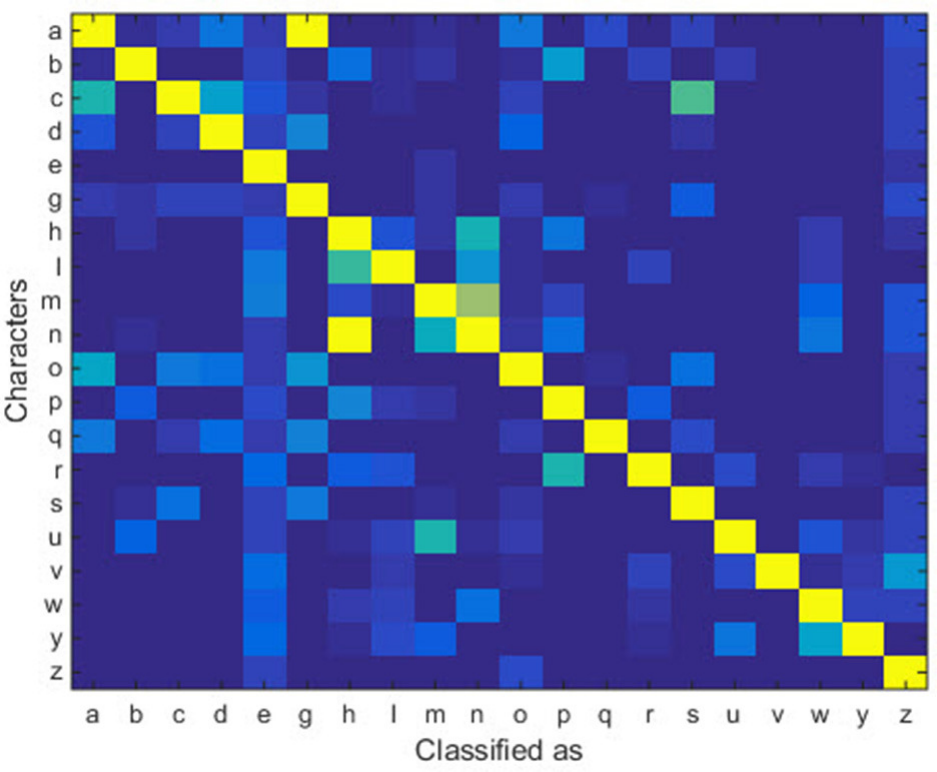
Confusion matrix of UCI dataset.

With the help of the slow CTLSTM blocks, CTLSTM has better performance than traditional LSTM on multiple character trajectories classification on both cases. Figure 6 shows the real-time classification outputs. The red lines denote the output neuron activation corresponding to the correct class, and the blue lines mean the output neuron activation corresponding to the other classes. The activation of the correct class of CTLSTM goes up and never falls down in Figure 6. The neuron activation illustrated using pixels is shown in Figure 7. Brighter pixels express higher activation value, while the darker ones express lower value. X axis denotes the time axis while the Y axis is the activation of the corresponding neuron number. The neuron activation of the traditional LSTM layer is shown in the top part of Figure 7. Out of the 130 neurons in total, the 30 neurons are in the slow CTLSTM layer. The activation of the slow and fast CTLSM layer neurons can be seen in the middle part of Figure 7. As illustrated in Figure 7, we can distinguish the neuron activities of LSTM and CTLSTM. We can see that the neuron activity of LSTM is uniform for all the neurons. Whereas, the slower CTLSTM neurons can be seen to start its activations with a delay since the timescale is larger for those slow neurons. On the other hand the faster CTLSTM behaves similar to the LSTM since the timescale is 1 for these neurons as the case of LSTM. In the case of LSTM, the activations of all the neurons fires frequently, similar to the fast CTLSTM cell. However, in CTLSM, we can easily distinguish that the cells in the slow CTLSTM layer have slower activation than the ones of fast CTLSTM layer. This feature helps the model to become more stable in the real-time classification task. It would be more easily for a slow CTLSTM block to capture and hold an important dynamic feature than a fast CTLSTM (LSTM) block. The classification accuracy, the error curve of classification and prediction are shown in Figures 8–10, respectively. The structure illustrated in Figure 4 is also implemented for LSTM in order to conduct a fair comparison. We implemented a two layer LSTM with 100 + 30 LSTM blocks and compare it to CTLSTM. Similar to the classification error decreasing curve shown in Figure 9, the prediction error of CTLSTM decreases faster than LSTM as shown in Figure 10. The classification performance is shown in Table 2. The experiment results show that prediction helps both CTLSTM and LSTM to converge faster, and CTLSTM outperforms LSTM in both prediction as well as classification. The confusion matrix of CTLTM with prediction is shown in Figure 11. It can be seen that both algorithms have some difficulty in classifying similar pairs such as “*n*” and “*h*”, “*a*” and “*c*”, “*q*” and “*g*,” etc.

Furthermore, Table 3 shows the Wilcoxon signed-rank test to check the difference in performance between CTLSTM and LSTM. As illustrated in the results, CTLSTM outperforms LSTM significantly.

**Table 3:** Wilcoxon signed-rank test results for the UCI dataset.

	**Method 1**	**Method 2**	***Z***	^**^p
Offline	LSTM	CTLSTM	−2.803	0.005
Online	LSTM	CTLSTM	−2.803	0.005

The classification performance of CTLSTM shows significant increase in the comparison (

** *p < 0.01)*.

### Microsoft research cambridge-12 kinect gesture dataset

We use the Microsoft Research Cambridge-12 (Fothergill et al., 2012) dataset in this experiment. The dataset consists of sequences of human movements, represented as body-part locations, and the associated gesture to be recognized by the system. It included 594 sequences and 719,359 frames—~6 h and 40 min—collected from 30 people performing 12 gestures. In total, there are 6,244 gesture instances. Each sequence lasts about 900–3,000 frames. We use 100 fast with 30 slow blocks for CTLSTM structure. LSTM with 130 blocks is used for comparison. The average length of MRC12 dataset is about 1,000 frames. Thus, the time constant we used for slow blocks is set to 40. The motion files contain tracks of 20 joints estimated using the Kinect Pose Estimation pipeline. X, Y coordinates and the depth distances are recorded in the dataset. The body poses are captured at a sample rate of 30 Hz with an accuracy of about 2 cm in joint positions.

Similar to our previous experiment, we randomly select half of the dataset to be used for training and the other half is used for testing. The stopping point was chosen when the error did not decrease after 30 epochs. The dataset is normalized by using min-max normalization. The details of the 12 kinds of motions, and the sequences used for train and test are shown in Table 4.

**Table 4:** Dataset description of Microsoft Research Cambridge-12 (MRC12) dataset.

**Train and test sequences**				
	**Classes**	**Training set**	**Test set**	**Total**
**Iconic gestures**	Crouch or hide (G2)	25	25	50
	Put on night vision goggles (G4)	25	25	50
	Shoot a pistol (G6)	25	24	49
	Throw an object (G8)	25	24	49
	Change weapon (G10)	24	24	48
	Kick (G12)	25	24	49
**Metaphoric gestures**	Start music/raise volume (of music) (G1)	25	25	50
	Navigate to next menu (G3)	25	25	50
	Wind up the music (G5)	24	24	48
	Take a bow to end music session (G7)	25	25	50
	Protest the music (G9)	25	25	50
	Move up the tempo of the song (G11)	25	24	49

**Figure 12:**
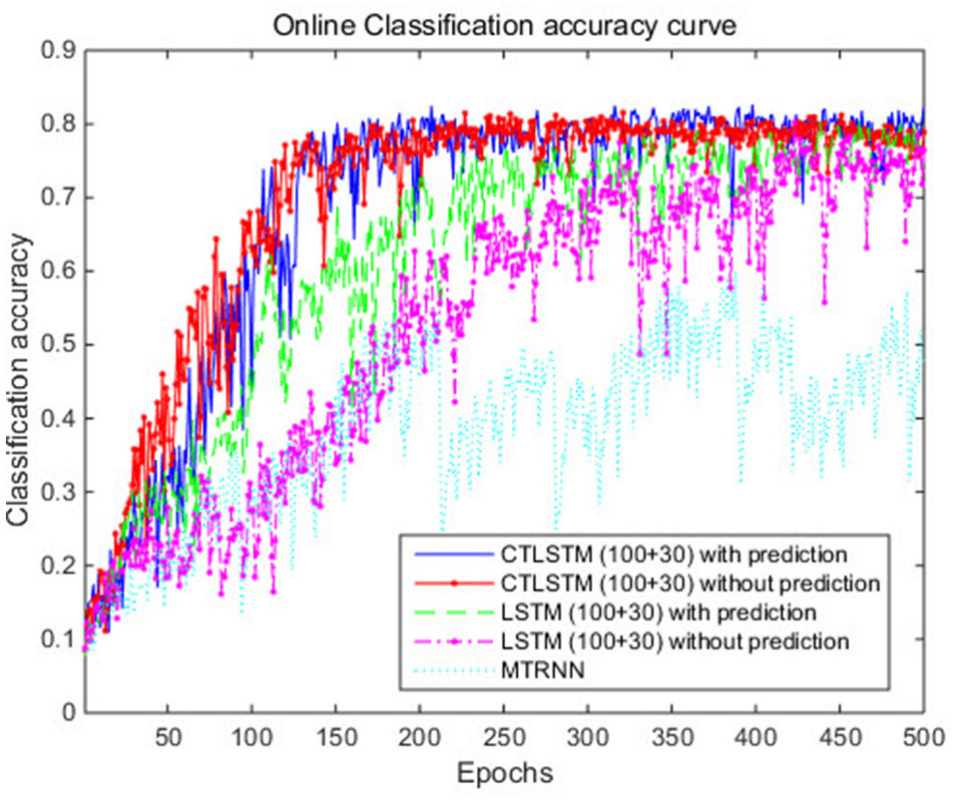
Classification accuracy (true positive) curve of CTLSTM and LSTM for Microsoft Research Cambridge-12.

**Figure 13:**
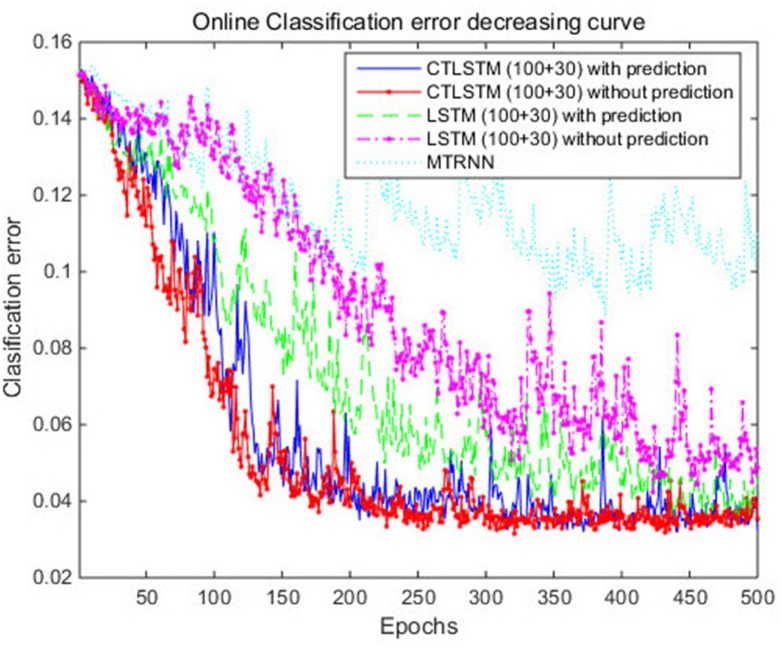
Classification error decreasing curve of CTLSTM and LSTM for Microsoft Research Cambridge-12.

We set the learning rate for both LSTM and CTLSTM to 0.00001. In both models, each block included one cell. Max pooling was used for classification decision in both models. The classification performance is shown in Table 5. CTLSTM shows better performance than LSTM. We also report the Wilcoxon signed-rank test in Table 6 and the results indicate the significance of the performance of CTLSTM over LSTM and MTRNN. The accuracy and the error curve of the training are shown in Figures 12, 13, respectively. Note that CTLSTM converges much faster and is more stable than LSTM. With the help of slow blocks, CTLSTM manages to outperform LSTM. We also compare our model to another neural recurrent model called Supervised MTRNN (Yu and Lee, 2015b). The neuron number, network structure and timescales used in Supervised MTRNN are the same as CTLSTM. We omit the result of Supervised MTRNN in the first experiment with UCI dataset because Supervised MTRNN did not converge over 1,000 epochs.

**Table 5:** Online classification results for Microsoft Research Cambridge-12 (MRC12) dataset.

**Real-time classification accuracy in %**				
**Classes**		**CTLSTM**	**LSTM**	**MTRNN**
**Iconic gestures**	Crouch or hide (G2)	73.78 ± 5.69	69.04 ± 7.68	31.88 ± 19.07
	Put on night vision goggles (G4)	86.69 ± 3.35	80.35 ± 6.49	65.68 ± 24.68
	Shoot a pistol (G6)	87 ± 4.2	79.01 ± 3.83	54.54 ± 25.89
	Throw an object (G8)	84.89 ± 4.61	79.17 ± 6.68	39.1 ± 25.88
	Change weapon (G10)	78.16 ± 4.54	74.13 ± 3.84	24.54 ± 11.45
	Kick (G12)	85.59 ± 4.61	86.77 ± 6.65	56.28 ± 17.74
**Metaphoric gestures**	Start Music/Raise Volume (of music) (G1)	82.46 ± 5.34	77.17 ± 5.28	49.43 ± 29.48
	Navigate to next menu (G3)	89.38 ± 1.52	91.36 ± 0.97	48.09 ± 18.43
	Wind up the music (G5)	73.03 ± 4.47	72.66 ± 6.69	35.3 ± 23.06
	Take a Bow to end music session (G7)	69.12 ± 5.97	76.62 ± 3.03	55.63 ± 17.8
	Protest the music (G9)	62 ± 6.65	61.28 ± 8.6	37.55 ± 14.12
	Move up the tempo of the song (G11)	86.95 ± 1.65	86.57 ± 1.96	37.95 ± 12.45
**Average**		**79.92** ± **1.45**	77.84 ± 1.71	44.66 ± 8.22

*Number in bold shows the best performance*.

**Table 6:** Wilcoxon signed-rank test results for Microsoft Research Cambridge-12 (MRC12) dataset.

**Method 1**	**Method 2**	***Z***	^**^p
LSTM	CTLSTM	−2.599	0.009
MTRNN	CTLSTM	−2.803	0.005
MTRNN	LSTM	−2.803	0.005

The classification performance of CTLSTM is shown to have a significant increase in the comparison (

** *p < 0.01)*.

**Figure 14:**
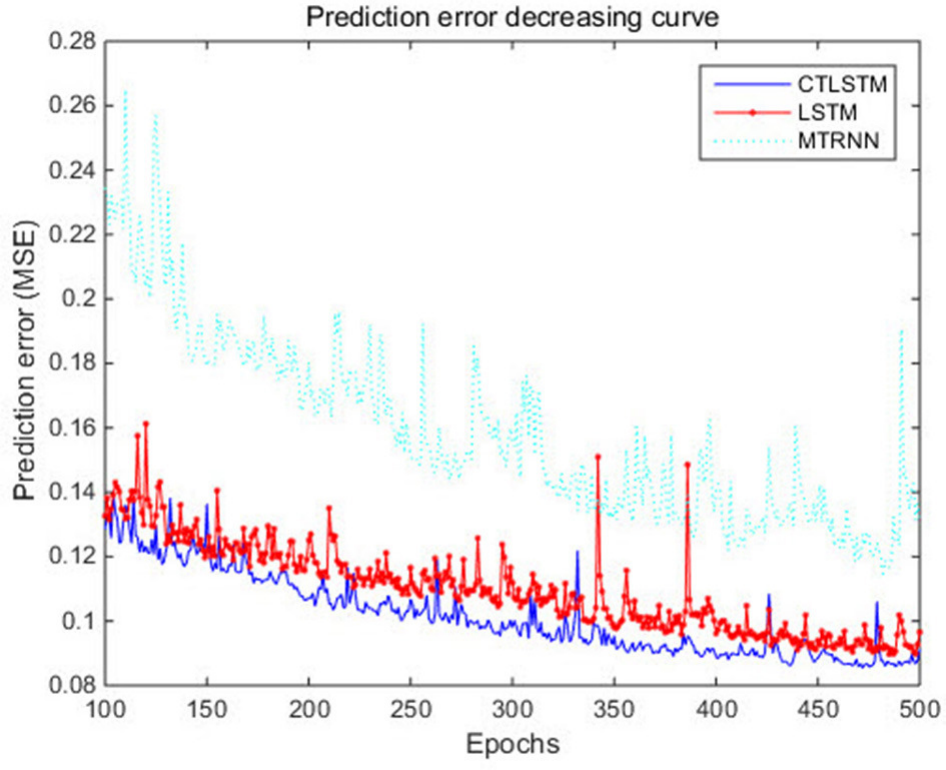
Prediction error decreasing curve of CTLSTM and LSTM for Microsoft Research Cambridge-12.

We also test the prediction performance of LSTM, CTLSTM and Supervised MTRNN in Figure 14 for the Microsoft Research Cambridge-12 dataset. Similar as the results shown in Figure 10, prediction error of CTLSTM decreases faster than LSTM.

### Human action dataset

**Figure 15:**
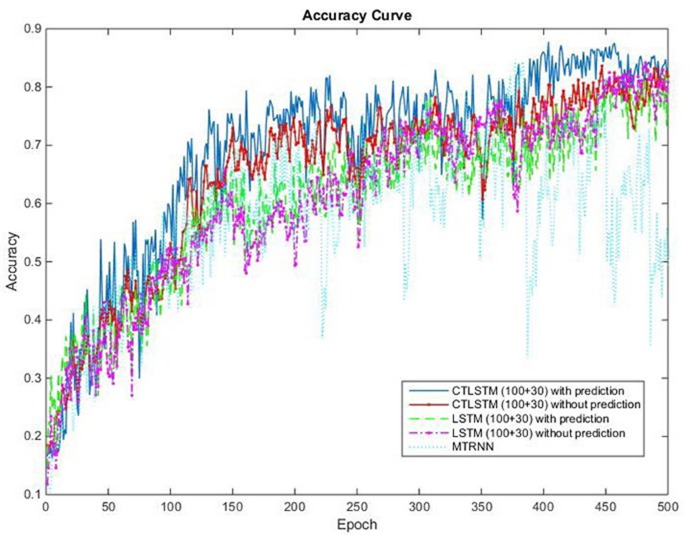
Classification accuracy (true positive) curve of CTLSTM, LSTM, and MTRNN for 10 kinds of actions.

**Figure 16:**
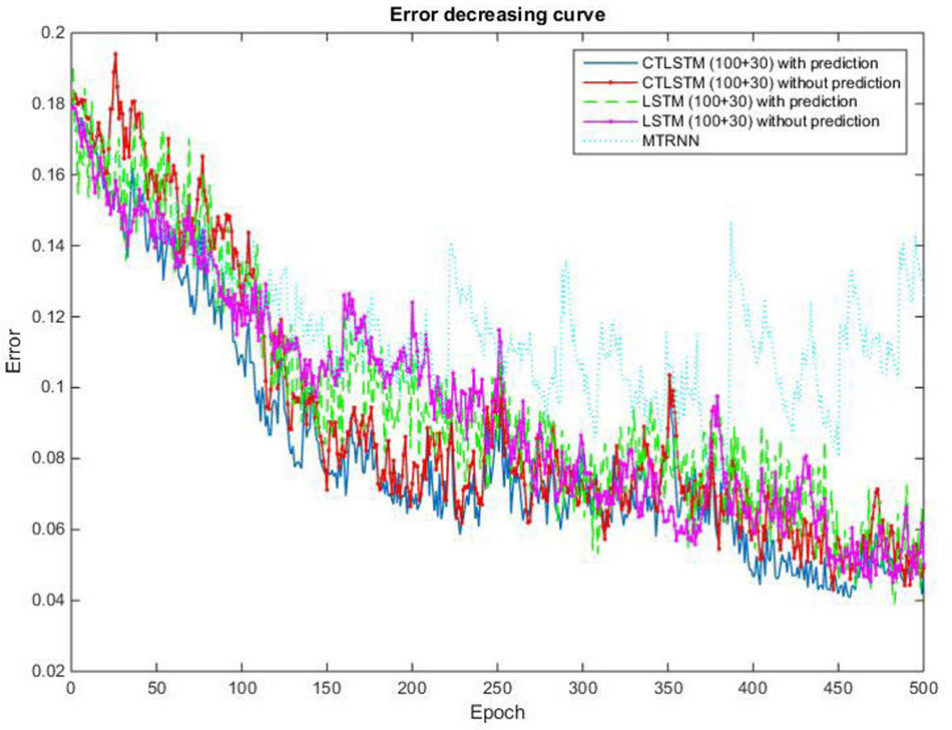
Classification error decreasing curve of CTLSTM, LSTM, and MTRNN for 10 kinds of actions.

This experiment is conducted on a custom dataset collected by us. It consists of long sequences of human movements, represented as body-part locations, and the associated gesture to be recognized by the system. The dataset includes 200 sequences and 200,000 frames—~2 h—collected using 10 volunteers performing 10 actions. Each sequence lasts about 1,000 frames. The details of this dataset and the sequences used for training and testing are listed in Table 7. The data contain tracks of 25 joints estimated collected using Kinect v2. X, Y coordinates and the depth distance are recorded in the dataset. The body poses are captured at a sample rate of 30 Hz with an accuracy of about two centimeters in joint positions. When recording the data, volunteers are free to do the specified action instances arbitrary number of times during the 1,000 frames. The parameters chosen for our dataset are the same with MRC12. The classification results are shown in Table 8. Table 9 shows the results of Wilcoxon signed-rank test and it can be seen that CTLSTM outperforms LSTM and MTRNN significantly. Figure 15 shows the classification accuracy of the training. The error curve of the training process is shown in Figure 16. Similar to Figures 8, 9, CTLSTM always converges faster than LSTM and Supervised MTRNN with the same block number and learning rate. As shown in Table 6, CTLSTM can be seen to perform well even in the case of longer sequences and outperforms the Supervised MTRNN and LSTM baseline models.

**Table 7:** Dataset description for 10 kinds of actions.

**Train and test sequences**			
**Classes**	**Training set**	**Test set**	**Total**
Standing	10	10	20
Eating noodles	10	10	20
Drinking	10	10	20
Clapping	10	10	20
Raising hand	10	10	20
Pointing	10	10	20
Bowing	10	10	20
Crouching	10	10	20
Punching	10	10	20
Kicking	10	10	20

**Table 8:** Online classification results for 10 kinds of actions.

**Real-time classification accuracy in %**			
**Classes**	**CTLSTM**	**LSTM**	**MTRNN**
Standing	90.64 ± 2.78	91.31 ± 3.98	90.33 ± 5.16
Eating noodles	81.58 ± 2.76	66.55 ± 3.7	71.6 ± 6.46
Drinking	79.63 ± 3.16	78.38 ± 4.45	73.89 ± 6.04
Clapping	65.32 ± 2.91	79.01 ± 5.26	95.05 ± 3.75
Raising hand	96.53 ± 2.4	85.83 ± 5.28	90.33 ± 4.07
Pointing	87.76 ± 2.82	77.67 ± 7.06	67.77 ± 6.15
Bowing	84.11 ± 2.97	86.87 ± 4.77	86.13 ± 4.99
Crouching	96.04 ± 2.58	94.07 ± 3.58	89.67 ± 5.27
Punching	75.53 ± 3.2	50.78 ± 7.27	35.38 ± 5.82
Kicking	85.9 ± 2.81	63.65 ± 7.78	38.4 ± 5.9
**Average**	**84.3** ± **1.43**	77.41 ± 1.91	73.86 ± 1.69

*Number in bold shows the best performance*.

**Table 9:** Wilcoxon signed-rank test results for 10 kinds of actions.

**Method 1**	**Method 2**	***Z***	^**^p
LSTM	CTLSTM	−2.803	0.005
MTRNN	CTLSTM	−2.803	0.005
MTRNN	LSTM	−2.803	0.005

The classification performance of CTLSTM can be seen to have a significant improvement in the comparison (

** *p < 0.01)*.

### Intention understanding dataset

We also perform the experiment using an intention understanding dataset collected by us. This dataset is more challenging and requires more capability to handle longer sequences because unlike the human action dataset described in the previous experiment, which consists of a single action in each class, this dataset consists of long sequences of multiple human actions in each intention class. The conditions of collecting the data are the same as the previous experiment. The data are collected using 10 volunteers performing two kinds of actions each for five different intention classes. The dataset includes 100 sequences and 200,000 frames. Each sequence lasts for about 2,000 frames. The sequences used for training and test are listed in Table 10. We compare the sequence length of the human action dataset and the intention understanding dataset in Table 11. As we can observe from the tables, in the intention understanding dataset, the sequence length of each class as well as the time duration of each class is double the human action dataset. The longer sequence data need a model which can capture the context well for longer period of time for intention understanding.

**Table 10:** Dataset description for five kinds of intentions.

**Train and test sequences**				
**Intention classes**	**Actions included**	**Training set**	**Test set**	**Total**
Having a meal	Eating + drinking	10	10	20
Fitness exercise	Standing + crouching	10	10	20
Appreciate	Clapping + bowing	10	10	20
Seeking attention	Raising hand + pointing	10	10	20
Aggression	Kicking + punching	10	10	20

**Table 11:** Comparison between human action and intention understanding datasets.

**Dataset property**	**Human action dataset**	**Intention understanding dataset**
Sequence length in each class	1,000	2,000
Frames per second	30	30
Sequence duration of each class in seconds	33.33	66.66

**Figure 17:**
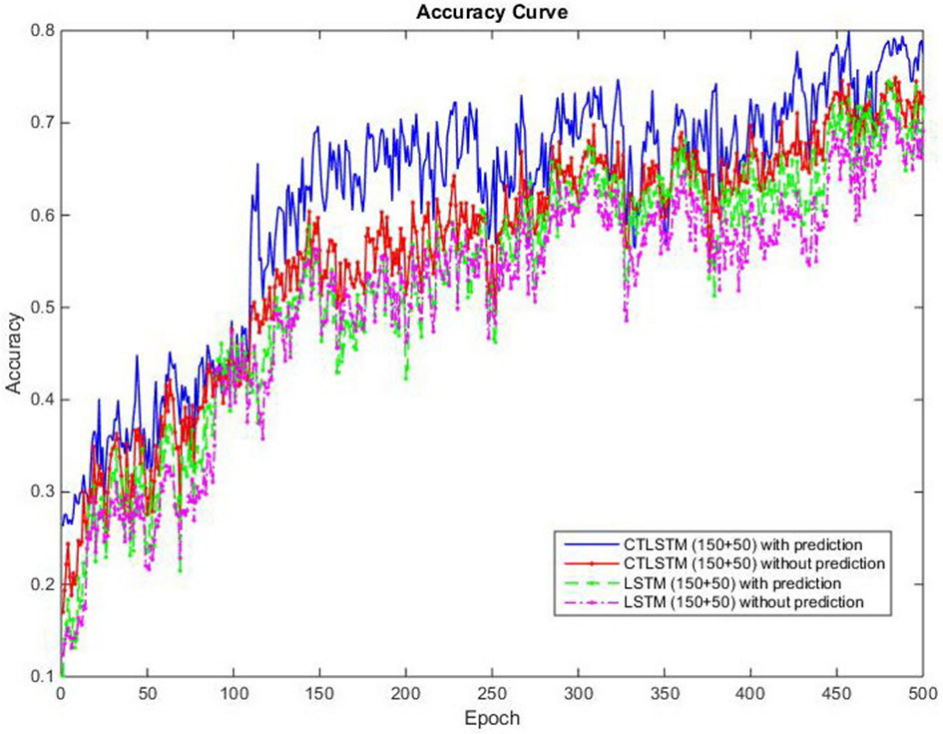
Classification accuracy (true positive) curve of CTLSTM and LSTM for 5 kinds of intentions.

**Figure 18:**
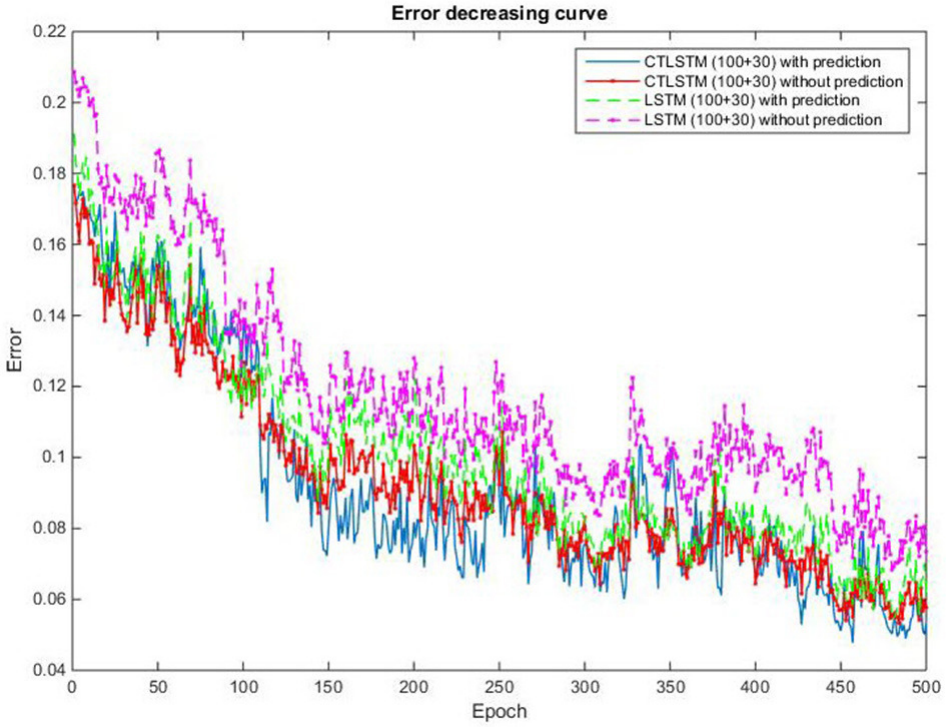
Classification error decreasing curve of CTLSTM and LSTM for 5 kinds of intentions.

We use 150 fast and 50 slow blocks for our CTLSTM model with a time constant of 40 for the slow blocks. In order to compare our model with the baseline, we train a two layer LSTM model with (150 + 50) blocks with one cell each. We set the learning rate for both LSTM and CTLSTM to 0.00001. The classification decision in both the models use max pooling. We omit the comparison results of Supervised MTRNN in this experiment because Supervised MTRNN did not converge even after 1,000 epochs of training. The classification results of the two models are shown in Table 12 and the classification accuracy curve is shown in Figure 17. The error decreasing curve of the training is shown in Figure 18. Table 13 shows the results of Wilcoxon signed-rank test to compare the performance between CTLSTM and LSTM. As the results illustrate, the performance of CTLSTM is consistent with the previous experiments as it converges faster compared to the LSTM model. The results also show that with the help of the timescales in the CTLSTM model to capture the dynamic context from the longer sequences efficiently, it is able to outperform the existing models, thereby making it the most suitable model for intention understanding tasks.

**Table 12:** Online classification results for 5 kinds of intentions.

**Real-time classification accuracy in %**		
**Intention classes**	**CTLSTM**	**LSTM**
Having a meal	82.2 ± 4.86	71.94 ± 4.98
Fitness exercise	87.53 ± 5.21	84.65 ± 6.18
Appreciate	75.88 ± 5.11	75.67 ± 6.76
Seeking attention	77.67 ± 5.94	62.59 ± 4.27
Aggression	66.22 ± 4.86	52.02 ± 6.67
**Average**	**77.9** ± **2.22**	69.37 ± 1.99

*Number in bold shows the best performance*.

**Table 13:** Wilcoxon signed-rank test results for 5 kinds of intentions.

**Method 1**	**Method 2**	***Z***	^**^p
LSTM	CTLSTM	−2.803	0.005

The classification performance of CTLSTM is significantly better than LSTM in the comparison (

** *p < 0.01)*.

## Conclusion and discussion

We developed the Continuous Timescale LSTM (CTLSTM) model inspired by the CTRNN and LSTM. Our proposed CTLSTM model benefits from the multiple timescales and is equipped to assign different work on different layers. CTLSTM is proved to have better performance on multiple classification tasks. We have shown the effectiveness of our proposed model in longer sequence understanding tasks and we argue that our model will be suitable for human behavior and intention understanding using deep learning techniques.

It should be noted that our model is quite different from the hierarchical multiscale RNN (Chung et al., 2017) and Multi-Timescale Long Short-Term Memory Neural Network (Liu et al., 2015). In their work, they define slow LSTM layer as a normal LSTM layer but with just a slower input rate. That is, not every frame is used as the input to the slow LSTM layer. In this case, the input of the slow LSTM layer is very important and the fast LSTM cells should capture the useful information and make sure the key information is transferred to the slow LSTM layer as inputs. Unlike their work, we define slow LSTM layer using an additional CTRNN activation where each frame is as the input of the slow LSTM layer.

The multiple timescales structure gives CTLSTM more capability to hold the information by improving the organization of the architecture and focusing on different task at different levels. LSTM has a special cell/block structure, which is able to hold important information for a long time if the forget gate is always closed. However, the gate opening time is still determined by BPTT. But BPTT itself may not have much ability to decide the temporal scale of the blocks in order to focus on different contexts. Thus CTLSTM, with the ability to guide the fast and slow blocks for different contexts is able to handle longer sequences efficiently compared to LSTM models.

The capacity of CTLSTM is highly correlated with the timescale constants. Theoretically, we can make one block (a very slow block) fire for longer period by assigning a large time constant (for example, 1,000). But a block with a large time constant would be difficult for training. Due to the restriction of Equation (23) large time constant would decrease the $\;\frac{\partial E}{\partial s_{c}^{t}}$. If $\frac{\partial E}{\partial s_{c}^{t}}\approx 0$, then $\frac{\partial E}{\partial u_{c}^{t}}$, $\frac{\partial E}{\partial u_{\phi }^{t}}$ and $\frac{\partial E}{\partial u_{l}^{t}}$ would also approach 0. So the weights would not be updated due to:

(24) $$\frac{\partial E}{\partial w_{ij}}=\frac{\partial E}{\partial u_{j}}y_{i}$$

This brings difficulty for slow neurons to get features from the input or other neurons. In our experiments, the timescale constant of the CTLSTM is set to 20 or 40 based on the dataset. In the future, we aim to solve this difficulty of setting the timescale constants by developing an adaptive mechanism for the timescales during the training process.

Fortunately, memory of RNN, which aims to further enhance the memory ability of RNN, already have been on focus in recent times (Sukhbaatar et al., 2015; Graves et al., 2017). We wish to make use of the advantages of timescales on the memory of RNN in our future work.

## Author contributions

All authors contributed equally and extensively to the work presented in this paper. ZY and ML designed the model and experiments. DM collected, analyzed, and interpreted the data. ZY developed the code and performed the experiments. ZY wrote the manuscript draft. DM and ML revised the paper.

### Conflict of interest statement

The authors declare that the research was conducted in the absence of any commercial or financial relationships that could be construed as a potential conflict of interest.
